# Evolving Landscape in Liver Transplantation for Hepatocellular Carcinoma: From Stage Migration to Immunotherapy Revolution

**DOI:** 10.3390/life13071562

**Published:** 2023-07-14

**Authors:** Silvia Cesario, Virginia Genovesi, Francesca Salani, Enrico Vasile, Lorenzo Fornaro, Caterina Vivaldi, Gianluca Masi

**Affiliations:** 1 Unit of Medical Oncology 2, Azienda Ospedaliero-Universitaria Pisana, 56126 Pisa, Italy; silvia.cesario2805@gmail.com (S.C.); virgi.genovesi@gmail.com (V.G.); e.vasile@ao-pisa.toscana.it (E.V.); l.fornaro@ao-pisa.toscana.it (L.F.); caterina.vivaldi@gmail.com (C.V.); gianluca.masi@unipi.it (G.M.); 2 Institute of Interdisciplinary Research “Health Science”, Scuola Superiore Sant’Anna, Piazza Martiri della Libertà 33, 56124 Pisa, Italy; 3 Department of Translational Research and New Technologies in Medicine and Surgery, University of Pisa, 56126 Pisa, Italy

**Keywords:** hepatocellular carcinoma, liver transplantation, immunotherapy, combination treatment, down-staging, bridging

## Abstract

Liver transplantation (LT) represents the primary curative option for HCC. Despite the extension of transplantation criteria and conversion with down-staging loco-regional treatments, transplantation is not always possible. The introduction of new standards of care in advanced HCC including a combination of immune checkpoint inhibitor-based therapies led to an improvement in response rates and could represent a promising strategy for down-staging the tumor burden. In this review, we identify reports and series, comprising a total of 43 patients who received immune checkpoint inhibitors as bridging or down-staging therapies prior to LT. Overall, treated patients registered an objective response rate of 21%, and 14 patients were reduced within the Milan criteria. Graft rejection was reported in seven patients, resulting in the death of four patients; in the remaining cases, LT was performed safely after immunotherapy. Further investigations are required to define the duration of immune checkpoint inhibitors, their minimum washout period and the LT long-term safety of this strategy. Some randomized clinical trials including immunotherapy combinations, loco-regional treatment and/or tyrosine kinase inhibitors are ongoing and will likely determine the appropriateness of immune checkpoint inhibitors’ administration before LT.

## 1. Introduction

Hepatocellular carcinoma (HCC) is the most prevalent form of liver malignancy [1] and the third leading cause of cancer deaths worldwide with a 5-year survival rate of around 18% [2]. The World Health Organization, based on year-on-year estimates, anticipates that more than 1.3 million people will die from liver cancer by 2040 [3]. Factors such as tumor burden, liver function and clinical condition influence treatment choice, as proposed by the Barcelona Clinic of Liver Cancer (BCLC) staging system [4]. Among the several therapies available, LT achieves the highest survival benefit for limited-stage diseases (BCLC-0 and A) [5]. Mazzaferro et al. demonstrated that LT is an effective treatment for those patients whose disease fell within the so-called Milan criteria (single lesion ≤5 cm or up to three lesions each ≤3 cm, without vascular involvement or extrahepatic spread), resulting in an overall improvement in the four-year survival rate of 75% [6]. The great efficacy of LT in HCC brought the progressive extension of its application criteria; however, for patients beyond the Milan criteria, 5-year survival after LT progressively decreases with nodule size and number, ranging from 75% to 35% [7]. These rates are still higher than those of loco-regional treatments (LRTs) alone by roughly 20% [8]. Consequently, one of the novelties of the last BCLC update (2022) is represented by the recognition of LT as potentially the best therapeutic strategy for a broader disease sub-set, stressing the need for a tailored treatment to obtain down-staging before LT. In detail, compared to the 2018 version, upfront LT is recommended for the following: (i) small multifocal HCC; (ii) a subgroup of stage B BCLC patients who meet the “Extended Liver transplantation” criteria, specific to each local institution [9]; and (iii) successfully down-staged patients (initially beyond the Milan criteria) treated with LRT, like transcatheter arterial chemoembolization (TACE), transarterial radioembolization (TARE), ablation and radiotherapy [8]. LRTs obtain approximately a 40% rate of down-staging to within the Milan criteria [10], and thanks to this approach, the survival rate after LT is reported to be comparable to those originally within the Milan criteria [11]. San Francisco (UCSF) [7], up-to-seven [12], Toronto extended criteria [13] and those from the University of California are the broadest institution-specific LT criteria.

The utilization of systemic therapies to reduce tumor burden in patients with BCLC-B HCC not initially amenable to curative treatment might represent a new tool in the box of pre-LT therapies; still, its opportunity is very debated. Immune checkpoint inhibitors (ICIs) have recently become a new therapeutic option for advanced HCC. The new challenge is combining ICIs with LT as a modality for down-staging or bridging.

In this review, we present the available data on ICIs as a down-staging therapy for HCC before LT and its rationale. We compare the described outcomes with those from traditional LRTs and display the perspective of novel approaches (e.g., ICIs combined with LRTs) being tested in ongoing clinical trials.

## 2. Current Strategies for Down-Staging

### 2.1. Loco-Regional Treatment

LRTs, including TACE, TARE, radiofrequency ablation (RFA) and radiotherapy, are commonly used in clinical practice to lower tumor burden before LT, to reduce tumor growth, prevent dropouts from waiting lists and increase survival after LT [14,15]. Moreover, the new EASL and AASLD guidelines encourage the use of LRTs for bridging and/or down-staging to improve the transplantation rate and reduce post-transplantation recurrence [16].

Optimal LRT choice depends primarily on their application goals. In case of bridging, RFA and TACE are recommended when the waiting time exceeds 6 months [17,18].

#### 2.1.1. RFA

RFA represents a valid alternative to surgery in BCLC-0/A HCC. Data from the European Liver Transplant Registry showed that RFA had the highest 5-year OS (80.9%) after LT compared to chemoembolization (67.6%) and no bridging or no down-staging LRTs (65.8%) [19]. Although RFA before LT can cause adhesions and inflammatory lesions, perioperative morbidity and mortality rate were not higher [20]. In the bridging-to-LT scenario, RFA reported a high rate of pathological complete responses in explanted livers compared to other LRTs, ranging between 62% and 71% depending on the series analyzed [18,21]. This effect depends on two fundamental factors: (a) nodules’ dimension, with a higher rate of response in lesions <2 cm, and (b) distance from hepatic vessels, because of the typical “heat sink effect” of this procedure [21]. Despite these promising results in terms of bridging, RFA does not seem to be the best strategy available for patients needing down-staging, due to a lack of evidence in this setting.

#### 2.1.2. TACE

TACE is a minimally invasive technique with a permanent curative effect and easy implementation which is recognized as the standard treatment for intermediate-stage HCC [16,22]. The down-staging rate is 48% and the 5-year OS ranges between 25% and 77.6%. There is a reduction in the dropout rate of 3–13%, especially when the expected waiting time for LT exceeds six months [17,23]. The ORR of TACE for BCLC-B HCC is approximately 52% and there is a correlation between the response to pre-LT TACE and HCC recurrence and OS [22,24]. There is debate regarding the safety of LT after TACE. Although appropriate TACE does not increase the risk of the transplant procedure, some studies have reported a high incidence of hepatic artery thrombosis and retransplantation after TACE [25]. Furthermore, it is not possible to establish whether it is preferable to use conventional TACE (cTACE) or DEB-TACE; however, DEB-TACE shows better tolerance and better long-term tumor control after complete pathological response [26].

#### 2.1.3. TARE

The updated AASLD guidelines suggest TARE as an alternative conversion strategy to TACE, especially in cases of portal vein thrombosis or tumor burden, and TARE can achieve a regression rate of up to 25–50% [27,28]. There is no difference in terms of efficacy and down-staging rate (both less than 80%) in the comparison between TARE and TACE as reported by the recent MERITS-LT multicenter study. From the analysis of explanted livers, TARE statistically significantly improved local tumor control compared to TACE [26,29]. Long-term survival results confirmed the above statement: the overall survival (OS) at 1 and 3 years from the first down-staging procedure was 92.5% and 73.0%, respectively, with no significant difference found when TACE was compared with TARE [29].

### 2.2. Systemic Treatment

The opportunity to explore systemic therapy as a down-staging strategy derives from the significant advances made in the pharmacological landscape of HCC. Since sorafenib’s approval in 2008, many promising drugs, including other tyrosine kinase inhibitors (TKIs) and ICIs, have improved response rates. In detail, if sorafenib granted an ORR of 2–3% [30], lenvatinib showed non-inferiority to sorafenib along with a greater objective response rate (ORR) of 40.6% [31]. Both registrative trials were not designed to evaluate the use of TKIs for down-staging and/or conversion, so information about the number of patients evaluated for curative treatments is missing.

ICIs have revolutionized the management of HCC after TKIs; however, single-agent ICIs, such as nivolumab, pembrolizumab and tislelizumab, reported an ORR of less than 20% due to primary resistance mechanisms against these strategies [32,33,34,35]. Combination therapies of anti-PD-1 and anti-angiogenic and anti-PD-1 and anti-CTLA4 exhibit a more promising anti-tumor efficacy. In the group of anti-PD1 plus anti-angiogenetic treatment, atezolizumab and bevacizumab induced an ORR of 30% [36]. Similar results were found with pembrolizumab plus lenvatinib and camrelizumab plus rivoceranib, with an ORR greater than 25% [37,38]. As for ICI doublets, a combination of anti-PD1 and anti-CTLA4, durvalumab and tremelimumab showed an ORR of 24% [39]. In CheckMate 040, a phase 1/2 trial, nivolumab plus ipilimumab, results in a 30% rate of objective responses [32]. No down-staging or conversion rates are reported in the results of the aforementioned trials.

From these promising activity results, ICI-based regimens have been postulated as even more advantageous options in terms of down-staging than LRT. Moreover, in addition to their greater ORR rates and their role in tumor immunity, ICIs seem to be important for transplantation immunity.

#### 2.2.1. LRT and TKI Combination

Data about the combination of TACE and sorafenib are conflicting: in 2020, the TACTICS trial, TACE plus sorafenib, showed PFS and OS benefit as compared with TACE alone. Although the ORR is not reported, the combination showed better survival as compared to TACE alone, especially in patients outside the Milan and up-to-seven criteria; subsequent treatment was received in 58.8% of patients in the TACE plus sorafenib arm, but no LT was performed [40]. While unsuccessful in confirming the above results, several other randomized trials, such as SPACE, indicated an ORR of 35.7% for the combination strategy [41,42]. TACE-2 enrolled 399 UK patients with unresectable HCC to receive sorafenib/placebo plus TACE: it did not show differences in its primary endpoint, which was mPFS, between two arms [42]. The SPACE trial enrolled patients with intermediate-stage HCC to DEB-TACE plus sorafenib or placebo; time to progression (primary endpoint) was similar between two arms [41].

The SORAMIC trial examined the association of TARE and sorafenib in 424 advanced HCC patients, resulting in no survival benefit from the combination. In subgroup analysis, patients with a tumor burden of the up-to-seven criteria could benefit from the combined use of TARE and sorafenib [43].

Regarding newer strategies than sorafenib, there is little knowledge about the association between TACE and lenvatinib. Recently, Ding et al. showed a favorable time to progress and ORR of TACE plus lenvatinib over the same LRT plus sorafenib, which reached a remarkable 53%, especially in patients with PVTT [44]. The LAUNCH trial analyzed the combination of lenvatinib + TACE vs. lenvatinib alone in 338 advanced HCC patients and reported a significant improvement in survival, with an ORR of 54.1% in the lenvatinib + TACE group (vs 25.0% lenvatinib) and, above all, 16% of patients treated with levatinib + TACE underwent curative treatment [45].

#### 2.2.2. Combination of LRT and Immunotherapy

Recently, combination strategies have been evolving toward LRT and immunotherapy’s association.

The studies reported below are rather heterogeneous, as not all provide the association between ICIs and LRT in terms of sequential strategy, but some propose the administration of ICIs as a post-LRT maintenance.

In 2022, Sangro et al. presented data regarding the phase II trial NASIR-HCC, enrolling patients with unresectable HCC to receive TARE followed by nivolumab, with a promising ORR of 38.1% in all comers and 38.7% in patients with BCLC-B2 or higher [46]. A recent trial presented at ASCO GI 2023 demonstrated a promising clinical activity of the combination of pembrolizumab plus Y90-TARE in HCC patients with a multifocal disease or MVI, with an ORR of 30.8% [47]. A similar phase II trial, assessing the safety and efficacy of atezolizumab plus bevacizumab after Y90-TARE, is still ongoing [48].

Regarding the association of TACE plus ICIs, the IMMUTACE trial showed promising response rates from the combination of TACE plus nivolumab in intermediate-stage HCC (ORR 71.4% and 16.3% of complete response) [49], but no data are currently available in the advanced setting.

While to date there are no data on down-staging following LRT and ICI treatment, immunotherapy before LT will be discussed extensively in the following paragraphs.

## 3. Ongoing Trials with LRT and ICI-Based Systemic Combinations

Of particular interest is the triplet multimodality therapy based on combining LRT (mainly TACE) with both TKIs and ICIs to pursue down-staging and conversion in HCC patients with no extrahepatic spread.

The rationale for combining these strategies lies in the following factors: (1) anti-PD-1 antibodies block inhibitory signals given by the interaction of PD-1 with its ligand, activating an immune response against tumors; (2) TACE induces devascularization of HCC and releases tumor-specific antigens [50,51]; and (3) targeting VEGF1–3, FGFR1–4, PDGFR a, RET and KIT, TKIs inhibit the pro-neoangiogenic and immunosuppressive effects of the tumor microenvironment [50,52,53]. Nonetheless, the hypoxic microenvironment resulting from LRT eventually promotes the secretion of HIF-1α, bFGF and VEGF, resulting in angiogenesis, tumor recurrence and metastases.

The first piece of evidence derived from retrospective trials enrolling unresectable and/or TACE refractory HCC (Table 1) is the following: in these cohorts, the response rates of the combination of ICIs, TKIs and LRT ranges between 42% and 77%, with better survival with respect to doublet therapy. Moreover, three association studies between TACE, lenvatinib and ICIs reported promising conversion rates to surgery with radical intent (range 26–53%) [52,54,55].

Looking prospectively to these results, they could lead the way to an LT approach in highly selected patients according to the obtained response to trimodal treatment.

## 4. ICIs in Solid Organ Transplantation: Strategy Rationale and Lights and Shadows of Previous Experiences

ICIs have revolutionized the algorithm of several malignancies: initially, ipilimumab was approved for metastatic melanoma by the US Food and Drug Administration (FDA) [64], and subsequently, ICIs have been indicated in other cancer types, including HCC.

Studies involving ICIs did not enroll patients with solid organ transplantation (SOT) and data regarding the safety and efficacy in this patient subset are still missing [65].

Furthermore, SOT patients have a twofold higher risk of tumor compared with the general population [66], with a higher prevalence of melanoma and other skin cancers, anogenital cancers and non-Hodgkin’s lymphomas [67]. Accordingly, cancer represented the second leading cause of death in these patients [68], probably because of maintaining allograft tolerance with immunosuppressive drugs [69,70] and the less aggressive cancer treatments they are given due to their medical history [71].

Graft rejection incidence has been investigated through different case reports and preclinical evidence in ICI-receiving patients. The blockade of PD-1/PD-L1 or CTLA-4 pathways mediates acute graft rejection. The main histological features of graft rejection include T-cell infiltration and inflammation of the bile duct and endothelial systems [72]. The PD-1/PD-L1 co-inhibitory pathway is required for graft tolerance, as reported from mouse models [73]. There is an emerging consensus on PD-L1 expression as a potential biomarker for outcome in patients with SOT receiving anti-PD1 therapy. Yin et al. collected 28 cases from individual reports/series of liver transplant patients who received ICIs at a later stage; PD-L1 expression was tested in 7 patients. PD-L1 expression was positive in four patients, and graft rejection occurred in all four cases (100%). In another case series, five patients received post-transplant anti-PD1 toripalimab therapy after testing the absence of PD-L1 expression in their respective grafts. No graft rejection occurred in these patients. Therefore, one possible hypothesis is that high graft PD-1 expression is predictive of sensitivity to the PD-L1/PD-1 inhibitor and, consequently, has a higher risk of graft rejection than a PD-1-negative graft [74,75].

From this perspective, testing the graft expression of PD-L1 could be helpful in the clinical management of this not-so-infrequent subgroup of patients; in fact, the absence of PD-L1 expression in organ donors appears safe for graft tolerance before ICI therapy.

The PD-1 pathway appears to play a more critical role during graft immune tolerance than the CTLA-4 pathway [76,77,78]. Support for this theory came from the experience of Blazar et al., who proved that the risk of graft versus host disease (GVHD) increased with the anti-PD-1 antibody compared with the CTLA-4 blockade and that the combination of anti-PD-1 and anti-CTLA-4 is responsible for more severe GVHD than monotherapy treatment. In fact, in their six mice models that underwent bone marrow transplant, they observed that both CD4 + and CD8 + T cells were downregulated by the PD-1 pathway: this conclusion was derived from the use of different approaches to block the PD-1 pathway, including the well-known anti-PD-1 mAb. Moreover, they confirmed that there was an increased release of proinflammatory cytokines, particularly IFN-γ, after PD-1 pathway blockade that is likely associated with GVHD lethality [79].

Immunosuppressant protocols represent a key aspect in SOT and they are subjected to periodic updates. Calcineurin inhibitors (CNIs), including tacrolimus and cyclosporine, changed allograft survival; on the other hand, these drugs also have the strongest evidence of increased tumor risk [80]. In a pre-clinical study using non-invasive adenocarcinoma cell lines by Hojo et al., the exposure to cyclosporine led to invasive behavior with the development of metastasis. The authors also showed that cyclosporine induced the acquisition of invasive behavior by transforming growth factor beta (TGFβ) products of tumor cells [80,81]. Also, tacrolimus reported similar effects [82]. Dantal et al. observed that the tumor incidence in renal transplant patients treated with cyclosporine could be dose-dependent [83].

Conversely, rapamycin and sirolimus, Mammalian Target Of Rapamycin (mTOR) inhibitors, have been reported to prevent tumor growth and progression in animal models [84]. mTOR inhibitors reduce vascular endothelial growth factor (VEGF) levels [85]. Consistently, Kauffman et al. reported a lower incidence rate of any de novo malignancy in sirolimus/everolimus groups (0.6%) compared with cyclosporine/tacrolimus (1.8%) in patients with a kidney transplant [86]. The introduction of ICIs in the SOT scenario will likely lead to remodulation of the intensity of immunosuppressant strategies; however, specific evidence is still lacking.

Another weakness in the knowledge of ICIs’ role in SOT is the limited robustness of the available literature: the majority of data emerge only from case reports or small series involving different clinical scenarios. Abdel-Wahab et al. published an interesting mono-institutional experience from MD Anderson Cancer Centre in 2019. They retrospectively collected data from 39 patients who received ICIs after SOT. In their cohort, the majority of patients had advanced melanoma (62%) and received anti-PD-1 agents (37.7%). The most frequently transplanted organs were the kidney (59%), liver (28%) and heart (13%). The median time between initiation of ICIs and SOT was 9 years (range 0.92–32 years), and 51% of patients had pre-emptive modification of the baseline immunosuppressive regimen before ICI initiation. Overall, graft rejection after ICIs occurred in 16 patients (41%) and the median time to graft rejection was 21 days (95% CI 19.3–22.8 days). Graft loss occurred in 40% of cases (12 of the 30 patients, 40%) treated with anti-PD-1 and 36% (5 of the 14 patients) treated with anti-CTLA-4. Rejection occurred more frequently in patients receiving single-agent prednisone (≤10 mg/day) at the beginning of ICIs than those receiving single-agent CNIs (78% vs. 11%, respectively). No differences were observed in the time between SOT and initiation of ICIs in patients with or without allograft rejection. The most common mechanisms of rejection identified via liver biopsies were the following: (1) acute rejection (75%) and (2) complex acute and chronic rejection (25%). Five patients who started ICIs at a median of 16 years (range 5–25 years) after SOT had a T-cell-mediated rejection; in the other five patients, who started ICIs at a median of 5 years (range 1.9–19 years) after SOT, a combination of cellular- and antibody-mediated rejection was reported. Four patients showed positive PD-1/PD-L1 expression at immunofluorescence analysis conducted on transplantation liver biopsies. Eighteen patients died (46%) because of allograft rejection or its complications. Median OS was 5 months (95% CI 1–9 months) for patients who had allograft rejection, compared with 12 months (95% CI 8–16 months) for those who had no rejection (*p* = 0.03). There were no differences in the median OS between patients receiving different immunosuppressive regimens at ICI initiation [65].

Another interesting analysis was conducted by Manohar et al. in 2020, who identified 44 kidney transplant patients who received ICIs after SOT. A total of 68% of them had melanoma, 11% lung cancer, 11% squamous skin carcinoma, 5% Merkel carcinoma, 2% urothelial and 2% duodenal cancer. A total of 36 patients received ICIs in monotherapy, of which 15 (34%) cases received anti-PD-1. Eighteen patients (41%) had an acute rejection of the kidney allograft. The median time from ICI initiation to acute rejection diagnosis was 24 (range, 10–60) days. Also, in this analysis, cellular rejection (33%) and mixed cellular and antibody-mediated rejection (17%) are confirmed the most common mechanisms of allograft rejection. Eventually, allograft failure occurred in 83% (15/18) of patients and eight patients died subsequently. Approximately half of the patients were treated with a CNI, mycophenolate mofetil and a low-dose steroid. Interestingly, 11 patients were tapered down to steroids alone for graft preservation at the time of checkpoint inhibitor initiation. At the latest follow-up (length not reported), 18 patients were alive. Fifteen patients maintained response to treatment (four in complete response, four in partial response and seven in stable disease) despite half of them having allograft failure [80].

DeLeon et al. reported the efficacy and safety of anti-PD-1 therapy in LT patients in a retrospective study. Seven patients with a previous LT received ICIs: five for HCC and two for melanoma. Rejection occurred in two of seven patients (28.6%) as an early event with a median time of 24 days. Regarding tumor response to ICI treatment, one patient showed complete response (CR) while three patients experienced progressive disease (PD). Three patients discontinued therapy before rescheduling assessments. Two of five patients with available tissue had tumor PD-L1 expression in the allograft and both developed rejection [87].

Rammohan et al. presented an interesting clinical case in 2018: a 57-year-old man with multifocal HCC who underwent living donor LT after multiple cycles of TACE and RFA. Following a diagnosis of metastatic disease, his immunosuppression was modified by adding mTOR inhibitors (tacrolimus dose decreased to maintain a blood level of 2–3 ng/mL), and he received sorafenib for one year. For progressive disease, he started pembrolizumab along with sorafenib. After 3 months, a CT scan showed an excellent response to the combination with a complete radiological response. After ten months of starting therapy, he remained well on pembrolizumab and sorafenib with no radiological evidence of disease [88].

To date, given the high rejection rate and fatal complications, post-transplant immunotherapy is contraindicated and is not a standard strategy.

## 5. Immunotherapy in LT: Down-Staging and Bridging

The use of ICIs as bridging or down-staging therapies before LT has been described in 17 case series. Literature data are generated from descriptive case studies and add up to 43 patients from different institutions. Despite limitations posed by this numerosity, we hereafter attempt to aggregate these data for the sake of their interpretation (Table 2). Male gender was the most frequent and HBV/HCV was the leading etiology for HCC. Of the available data, 19 patients exceeded the Milan criteria at baseline and received other treatments before ICI. Previous treatments included LRTs in 21% of patients and TKIs in 16% of patients. Of these, in 10 case series, a total of 30 patients received ICI monotherapy and nivolumab was the most used. In the remaining seven, a total of 13 patients received combination therapy including ICIs. Although the duration of therapy was unevenly reported across the studies, seven studies displayed a median treatment duration of 9 cycles (range 1–34). The longest ICI treatment lasted two years. The washout period between the ICI’s last dose and LT ranged from 1 day to 10 months. Even though the assessment of the best overall response rate was not specified in all studies, the ORR was approximately 21%. Globally, these experiences reported the down-staging within the Milan criteria of 14 patients, granting them the possibility of LT.

Despite the high response rate with ICIs, LRT was applied to stabilize lesions or to consolidate a response in five patients [89,90,91,92]. Patients’ outcomes varied considerably in each study. A successful graft, defined as the absence of acute rejection, was performed in 74% (32/43) of patients [74,91,92,93,94,95,96,97,98,99]. Xenograft pathology revealed almost complete (>90%) tumor necrosis in seven patients [98,99]. For instance, Schmiderer et al. described a case of HCC BCLC stage C (portal vein invasion) who underwent LT after 6 months of treatment with atezolizumab + bevacizumab. Histological examination showed a complete histological response [98,99].

In terms of safety, 7% (3/43) of patients had mild rejection which was successfully treated by modifying their immunosuppressant scheme. Eight patients (out of 43) suffered graft rejection between 20 h and 14 days after LT and four of them died as a result of this complication. Nordness et al. described a patient who, after 2 years of nivolumab, received the final dose 8 days prior to LT and developed a transplant rejection. Chen et al. reported a case of acute graft rejection in a patient who received pre-transplant treatment with toripalimab. Specific to this case, on the 33rd hour after LT, liver function deteriorated, and the patient received continuous renal replacement therapy and plasma exchange treatment. However, the patient developed multiple organ failure. In the other cases, a worsening of liver function has also been described. In the single case of Nordness et al., high doses of methylprednisolone and rabbit antithymocytic globulin (rATG) were given without improvement in liver function tests. Dave et al. administered rATG to three patients, but only one of these had biopsy-proven rejection. Interestingly, Chen et al. reported that PD-L1 expression in preimplant donor liver tissue was negative but turned positive in postimplant tissue. This confirms that ICIs could lead to failure of the transplant attempt to reach “immune escape” by expressing PD-L1, which results in a lethal acute rejection response [89,90,98,100,101]. In one case (out of eight who developed graft rejection), salvage of the graft was possible with rATG, a high dose of steroids, intravenous immunoglobulin and rituximab [98].

Among eight patients who experienced graft rejection, three patients underwent re-transplant [90,98,102]. Dehghan et al. had the first case of rescue liver re-transplantation after loss of the first allograft following pre-transplant nivolumab. The ICI was administered for 15 months and LT was performed 5 weeks after discontinuing nivolumab. On postoperative day (POD) 10, the patient had a fever and increased transaminase levels. The patient underwent re-transplantation without recurrent graft loss after 18 months follow-up. Schickel et al. described a similar case that received a total of 18 months of nivolumab with the last dose 5 weeks before LT; transaminases increased on POD 16 and liver biopsies revealed an acute cellular rejection with sub-massive hepatic necrosis that led to re-transplant on POD 34. At 38 months post-transplant, the patient had a stable graft. Also, Dave et al. described a case of re-transplant, but no further information is available.

In conclusion, both mild and severe acute rejections were reported. A total of 8 of the 43 patients developed severe acute rejection. The most frequently used drugs were high-dose steroids and rATG. Dehghan et al. and Schnickel et al. described two different patients who developed acute rejection with high levels of donor-specific antibodies (DSAs). Compared to transplantation of other solid organs, the liver is quite immune-tolerant and most LT centers still do not account for human leukocyte antigen (HLA) matching in their allocation algorithms. DSAs are recipient-formed antibodies that can bind to HLA in the donor organ, causing damage to the graft. Preformed DSAs exist prior to transplant when the recipient has been exposed to a variety of non-self HLAs, whereas de novo DSAs form after transplantation in response to the new donor organ’s HLAs. Most preformed DSAs clear spontaneously early after transplant. In patients with persistent DSAs (preformed or de novo), there is a higher risk for overall rejection [103,104]. The use of ICIs before LT could change the immune tolerance characteristics of the liver; consequently, patients receiving ICIs before LT may benefit from screening for DSAs before and after LT. Also, the dosage of DSAs could be useful to evaluate the efficacy of high-dose immunosuppressive therapy during acute rejection. Interestingly, both Dehghan et al. and Schnickel et al. performed re-transplantation after observing the reduction in DSA levels, which may have contributed to the favorable outcome for the two patients.

Overall, the death rate from rejection was 7% (3/43). Follow-up data and long-term outcomes are only partially available for 33 patients. Although there were different periods of follow-up (range: 7–38 months), no allograft rejection occurred in most of the patients. Schnickel et al. reported that one patient died 2 days after his last follow-up visit from a cardiac arrest with a functioning graft and normal liver tests. Chen et al. reported two patients who developed HCC recurrence: one progressed in the liver, bone and lungs after 7 months and the other experienced recurrence in the lungs after 3 months. Based on the data available from studies on ICIs before LT, the recurrence rate of HCC was 5% (2/43).

**Table 2:** Previous case series of immunotherapy before liver transplantation.

Author [Citation]	Number of Patients (N)	Gender (Male/Female) (N)	Etiology (HBV/HCB/NASH/Alcohol/Not Known) (N)	ICI	Duration	Milan in Post-ICI (IN/OUT) (N)	Other Treatments after ICI	Last Dose (Time)	Rejection (Yes/No) (N)	Time to Rejection	Outcome (No Recurrence/Recurrence/Death/Re-transplant/Solved with Medical Therapy) (N)
Abdelrahim, 2022 [93]	1	1/0	0/1/0/0/0	Atezolizumab + Bevacizumab	6 cycles	1/0	/	2 months	0/1	/	1/0/0/0/0
Schmiderer, 2023 [96]	1	1/0	0/1/1/0/0	Atezolizumab + Bevacizumab	6 months	1/0	/	6 weeks	0/1	/	1/0/0/0/0
Aby, 2022 [104]	1	1/0	0/1/0/0/0	Nivolumab	23 cycles	1/0	/	16 days	1 mild/0	9 days	0/0/0/0/1
Chen, 2021 [89]	1	1/0	1/0/0/0/0	Toripalimab + Lenvatinib	10 cycles	1/0	RFA	93 days	1 lethal/0	33 h	0/0/1/0/0
Chen, 2021 [94]	5	1/4	NA	Nivolumab	1–6 cycles	NA	/	7–122 days	0/5	/	3/2/0/0/0
Dave, 2022 [102]	5	5/0	NA	Nivolumab	NA	NA	/	105 days	2 lethal/3	/	3/0/1/1/0
Dehghan, 2021 [90]	1	0/1	0/1/0/0/0	Nivolumab	15 months	1/0	RFA	5 weeks	1 lethal/0	10 days	0/0/0/1/0
Lizaola-Mayo, 2021 [95]	1	1/0	0/0/0/1/0	Nivolumab + Ipilimumab	6 months	NA	/	8 weeks	0/1	NA	1/0/0/0/0
Nordness, 2020 [100]	1	1/0	0/1/0/0/0	Nivolumab	2 years	1/0	/	8 days	1/0	6 days	0/0/1/0/0
Peterson, 2021 [91]	1	1/0	0/1/0/0/0	Nivolumab	6 months	1/0	TARE	10 months	0/1	NA	1/0/0/0/0
Qiao, 2021 [74]	7	7/0	NA	Camrelizumab or Pembrolizumab + Lenvatinib	1–5 cycles	NA	/	1.3 months	1 mild/6	10 days	0/0/0/0/1
Schnickel, 2022 [98]	5	2/3	1/4/0/0/0	Nivolumab	8–18 months	NA	/	10 days–6 months	2/3	12–14 days	3/0/0/1/1
Sogbe, 2021 [97]	1	1/0	1/0/0/0/0	Durvalumab	15 months	NA	TACE	3 months	0/1	NA	1/0/0/0/0
Schwacha-Eipper, 2020 [92]	1	1/0	0/0/0/1/0	Nivolumab	34 cycles	1/0	MWA	6 weeks	0/1	/	1/0/0/0/0
Yin, 2022 [101]	1	1/0	1/0/0/0/0	Lenvatinib + Pembrolizumab	NA	NA	/		1/0	20 h after LT	0/0/1/0/0
Tabrizian, 2021 [99]	9	3/6	5/2/1/0/1	Nivolumab	2–32 cycles	3/6	/	Within 4 weeks	1 mild/0	NA	9/0/0/0/0
Solino, 2023 [103]	1	1/0	0/0/0/1/0	Atezolizumab + Bevacizumab	6 cycles	N	/	1 months	0	NA	1/0/0/0/0

TACE: transarterial chemoembolization, RFA: radiofrequency, MWA: microwave ablation, TARE: transarterial radioembolization, NA: not available, N: number.

## 6. Biological Markers to Detect LT Rejection

Despite advances in immunomodulatory therapies, acute rejection remains a significant complication after SOT. Diagnosis of rejection typically involves invasive biopsy sampling for histopathological analysis and non-invasive biomarkers for early detection are still an unmet need.

Cell-free DNA has emerged as a useful biomarker in multiple clinical settings. In oncology, the isolation of circulating free DNA allows for the studying of tumor molecular profiling in different malignancies. This gave rise to the notion of a “liquid biopsy” for diagnostic and management purposes. In SOT, genetic differences become fundamental. Except for an identical twin donor–recipient pair, it is possible to detect circulating free donor DNA (cf-dDNA) via minimally invasive blood sampling. This biomarker is found in all recipients. Based on the levels of cf-dDNA (low or high), graft integrity or graft injury/rejection can be detected, respectively [105,106]. Therefore, cfDNA could be used to prompt early adjustments to immunosuppressive therapy and prevent graft complications. Given the rising number of LT recipients, further donor-specific cf-dDNA research could be of high clinical impact; in fact, a large prospective trial is ongoing to validate particular cf-dDNA assays in LT [107]. Subsequently, randomized controlled trials could evaluate the impact of precision medicine compared to the standard of care after LT.

After LT, the immune microenvironment is subjected to changes that can be detected by flow cytometry. Recently, T-cell monitoring has helped to determine the effect of various T-cell subsets on transplant outcomes: Tregs have a critical role in establishing tolerance in LT recipients and both Foxp3 + CD25 high and CD4 + CD25 + CD127 low/- T cells have been identified as regulatory subtypes in humans, which might improve graft survival [108,109].

Han et al. demonstrated that active Treg concentrations on day 7 post-LT were considerably lower in subjects with biopsy-proven acute rejection than non-rejectors, together with a lower expression of Bcl-2, an anti-apoptotic molecule linked to the survival of Tregs. Multivariate Cox regression analysis confirmed Tregs on D7 as an independent risk factor [110].

Another proinflammatory player in graft rejection is Th17; it has been demonstrated that a balance between Th17 and Treg cell frequencies in recipients is essential to establish allograft. In the rejection group, the Th17/Treg ratio was significantly greater than in the stable one. Moreover, the percentages of Th17 and Treg cells in the peripheral blood of stable LT recipients at six months post-LT was significantly reduced, whilst the Th17/Treg ratio was comparable to the pre-transplant period [111]. This serological “marker” may be helpful during LT follow-up to predict the development of tolerance.

PD-1 plays an important role in the graft’s survival. During LT, immunological responses are inhibited and escape human surveillance due to the overexpression of immune checkpoint molecules; it has been demonstrated that PD-1 expression in CD8+ T cells was found to be significantly lower in acute rejection, suggesting that its downregulation could have a central role in this process [112].

Serological markers of allograft rejection are under study. A pilot study revealed a strong link between IL-6 levels and graft loss [113]. Moreover, rejection is predominantly mediated by CD4+ T cells, that can be induced by proinflammatory cytokines to produce remarkably higher levels of soluble fibrinogen-like protein 2 (sFGL2), another potential mediator of allograft rejection. Zhao et al. demonstrated that serum levels of sFGL2 and the percentage of CD4+ T cells in the peripheral blood were significantly increased in renal allograft recipients with acute rejection, compared with those with stable renal function [114].

All the abovementioned experiences constitute preliminary evidence of the crucial role of immune function monitoring during LT follow-up. Nonetheless, the reproducibility and efficacy of these postulated biomarkers need to be validated in large prospective trials.

## 7. Proposed Take-Home Messages from the Existing Literature on ICIs in LT

As a consequence of small sample size and selective bias of the available literature, determining the actual likelihood of lethal rejection for patients receiving ICIs as down-staging or bridging for LT still remains a special issue. For instance, no guidelines exist on ICI discontinuation timing, despite being an important factor to be considered.

A short interval between ICI infusion and transplant seems to increase the risk of acute rejection, although there are conflicting data in the literature; for example, Dave et al. reported graft losses in two patients who received ICI therapy < 90 days before LT, while Tabrizan et al. documented successful transplantation 1 and 2 days after the last nivolumab infusion. The minimal washout time is often set loosely based on the reported serum half-life for ICIs (Table S1, Supplementary Material). However, the interaction between ICIs and their pharmacological target can remain high for significantly longer: the serum half-life for nivolumab is 12–20 days, but a sustained occupancy of over 70% of PD-1 molecules on circulating T is observed for more than 2 months following a single infusion [115,116]. We hope that, in order to obtain a more precise estimate of the washout period, other potential parameters or markers are therefore studied.

Similarly, there is no agreement with respect to the duration of neoadjuvant immunotherapy as bridging for LT. In other solid tumor settings, patients received up to six months of neoadjuvant ICIs before surgery [117,118]. In a clinical study involving different solid malignancies, the reactivation of T cells peaked after only 1 week of pre-operative immunotherapy [119]. The enhanced immune activation may reflect an early time for response [33,39,120]; even after discontinuation of ICIs, antitumoral T cells in peripheral blood maintained a prolonged tumor response [121]. In study ML43352, the number of cycles of atezolizumab plus bevacizumab was established up to a maximum of eight during the LT waiting period for patients with HCC beyond the Milan criteria [122]. In KY2019-SHR-APA-ZJU, patients underwent camrelizumab treatment for at least 2 cycles in combination with apatinib [123].

Given the sustained occupancy of ICI pharmacological targets and the role of PD-1 in graft survival, it will be important to evaluate the interplay of ICIs in the immune microenvironment after LT. Currently, there is a partial understanding of the immune activation’s effects triggered by ICIs and immune modulation with immunosuppressive drugs. Achieving a balance between the two extremes is not easy and further comprehensive investigations are required.

Although rejection is an undesirable outcome in this setting, other immune-related adverse events (irAEs) can also occur. The incidence and onset of irAEs vary based on several factors: (a) the class and dose of the ICI administered, (b) the type of cancer and (c) patients’ related characteristics. In general, those receiving anti–PD-1 or PD-L1 monotherapy were exposed to a lower incidence of any-grade irAEs than anti–CTLA-4 agents, with combinations increasing the incidence, severity and onset of irAEs [124]. IrAEs have not been reported in the cited studies. ESMO guidelines reported that, for anti-PD-L1/PD-1, any treatment-related adverse events (AEs) were documented in at least 80% of patients and grade 3 to 4 toxicities were documented in 10–20% [125]. In the IMbrave 150 trial, grade 3–4 AEs occurred in 43% of patients in the atezolizumab and bevacizumab arm. Particular attention needs to be paid to hypertension and bleeding: grade 3–4 hypertension occurred in 18% and grade 3–4 bleeding occurred in 9% of patients treated with atezolizumab and bevacizumab [126]. The occurrence of these adversities can prolong or even end the transplant process, not only because AEs may render the patient ineligible for LT, but also because their clinical management may prolong the time to LT, resulting in progression of the malignancy and dropout from transplantation criteria.

The use of LRT reduces the chance of dropping out of the LT waiting list. Response to LRT—either used as bridging for Milan criteria or as down-staging for expanded criteria patients—has thus been proposed as a surrogate of favorable tumor biology [127]. Only 17% of patients after LRT did not undergo LT due to progression of HCC [128]. Despite improvement in the ORR, almost 40% of HCC patients did not achieve disease control with ICIs [129]. This high rate of the primary progressor could be responsible for dropout from the liver transplant waiting list. Currently, few biomarkers have been analyzed as predictors of ICI response; thus, we aimed to identify a predictive biomarker that could help in stratifying patients who benefit from ICIs as a down-staging treatment and, subsequently, for LT. Long-term follow-up to establish a true benefit in survival time of ICIs before LT is lacking.

There is no guideline or consensus on combining LRT with ICIs. Despite improvement in the response to newer systemic therapies, it is possible to integrate LRT to reduce viable tumors to meet the transplant criteria.

The recurrence rate of HCC ranges from 10% to 20%, with 50% of the patients classified as beyond the Milan criteria at the explant pathology evaluation [130] and the median time from LT to HCC recurrence is reported as 13 (range 2–132) months [130,131]. A total of 70% of recurrence cases are extrahepatic and 51% occurred in tumor burden classified as beyond the Milan criteria upon explanted liver analysis. Chen et al. reported two cases of early-onset HCC recurrence. Early recurrence occurs due to pre-transplantation staging inaccuracy, which fails to identify existing metastases or by the growth of circulating tumor cells’ resistance to ICIs. No information about late recurrence is available.

There is too little knowledge of the long-term security of ICIs and LT. The expression of PD-L1 in the graft protects against chronic rejection [132]. Although its prevalence has declined with the introduction of potent immunosuppressive therapy, chronic rejection still represents an important cause of graft injury. Whether prior use of ICIs may lead to chronic rejection is yet unknown.

### Ongoing Trials

Further investigations into down-staging strategies for LT are ongoing and comprise the combination of (1) ICI doublets (anti-PD-1 + anti-CTLA4); (2) ICI + anti-angiogenic (both anti-VEFG and TKI); and (3) LRT (such as TACE, HAIC, SBRT) + ICI and/or TKI.

At present, there are eight ongoing prospective trials investigating the impact of pre-liver transplant immunotherapy (Table 3). The PLENTY trial [133] is an open-label randomized trial testing safety and efficacy of the combination of pembrolizumab and lenvatinib in the treatment of HCC, when administered before LT. The primary outcome is the efficacy of the strategy studied as recurrence-free survival, while the secondary outcomes include safety in terms of the percentage of patients who experience adverse events, along with other activity measures such as ORR and disease control rate. Similarly, the open-label Dulect2020-1 [134] trial is testing the safety and efficacy of the durvalumab and lenvatinib combination in participants with locally advanced HCC before liver transplant and metastatic unresectable HCC. This trial has two primary outcome measures: progression-free survival or recurrence-free survival in patients with locally advanced HCC who underwent LT. Secondary outcomes include ORR, OS and adverse event rate. Interestingly, in Dulect2020-1, investigators planned to evaluate LT patients after 2 months of randomization and after 42 days of washout from the last ICI dose. The open-label ML43352 [122] enrolls patients with HCC beyond the Milan criteria who are transplant-eligible to receive atezolizumab plus bevacizumab. The ESR-20-21010 trial [135] is a single-arm, open-label, phase II trial aimed at evaluating the safety and efficacy of the double immune checkpoint combination of durvalumab and tremelimumab, before LT. Patients will be treated for up to 4 months. After a minimum of a 28-day washout, patients will undergo LRT per institutional standards, and, eventually, after a minimum 72-day washout, they will undergo LT. The phase I/II KY2019-SHR-APA-ZJU trial [123] aims to assess the primary effects and safety of camrelizumab plus apatinib for down-staging/bridging of HCC before LT; patients enrolled in this trial receive at least two doses of camrelizumab and stops it 5 weeks prior to LT. The results of these trials are highly awaited, not only to establish the activity and efficacy of the ICI strategy in the pre-LT setting but also to gather pivotal data on its safety. A central issue in this regard is the time lag between the last ICI administration and the LT procedure: while in the retrospective series these data are highly heterogeneous (ranging from 1 to 10 months as reported previously), the ongoing trials propose a shorter interruption of 4–6 weeks. Waiting for this evidence to be gathered, we believe that considering a washout of at least three half-lives (as suggested by the Investigator’s Brochure of drugs at higher risk of bleeding, such as anti-angiogenics) would be sensible. To tailor this empiric approach to the question, we strongly believe that dynamically testable circulating predictors of ICI activity and washout (such as plasma concentrations and immune cell subsets identified via flow cytometry, etc.) are highly needed to define the safest temporal window for this otherwise challenging approach.

The different strategies’ specific rationales rely on the abovementioned mechanisms.

## 8. Conclusions

According to the evidence examined in this study, the role of ICIs to bridge or down-stage HCC for LT is not well established. Nowadays, no biomarkers, tissue or liquid biopsies are available to help in selecting patients for immunotherapy and, subsequently, for transplant. The best approach to optimize patients’ administration with locally advanced diseases with excellent responses to systemic therapy and who meet transplant criteria is the evaluation of a dedicated multidisciplinary team (MDT). An MDT assessment pre-LT is essential to evaluate tumor burden, vascular involvement, liver function and physical status. Evaluation of response to ICIs and a combination of LRTs should be assessed by the MDT. Currently, there is no way to understand when to direct patients toward transplant after ICIs. Factors that may influence the MDT’s choice include time with stable disease on ICIs or the length of the overall treatment.

## Figures and Tables

**Table 1:** Retrospective trials evaluating the combination of LRT, ICIs and TKIs.

Name	Treatment	N	AFP (<400, ≥ 400)	CP A/B	MVI (Y/N)	EHS (Y/N)	BCLC A/B/C	ORR	PFS	OS	Conversion
Zheng 2021 [56]	TACE + Sor + ICIs	22	7/15	13/9	7/15	7/15	0/11/11	54.6%	16.3	23.3	NA
	TACE + Sor	29	8/21	18/11	8/21	13/16	0/14/15	34.5%	7.3	13.8	NA
Wu 2021 [52]	TACE + Len + ICIs	62	30/32	NA	34/28	6/56	6/21/35	77.4%	NR	NR	53.2%
Chen 2021 [54]	TACE + Len + Pembro	70	25/45	NA	NA	48/22	0/47/23	47.1%	9.2	18.1	25.7%
	TACE + Len	72	28/44	NA	NA	52/20	0/45/27	27.8%	5.5	14.1	11.1%
Liu 2021 [57]	TACE + Len + Camre	22	15/7	16/6	11/11	1/21	0/12/10	72.7%	11.4	23.6	NA
Cao 2021 [58]	TACE + Len + Sintilimab	52	34/18	46/6	19/33	21/31	0/13/29	46.7%	13.3	23.6	NA
Ju 2022 [59]	TACE + Apatinib + Camre	56	21/35	43/13	NA	NA	0/13/43	42.9%	NA	24.8	NA
	Apatinib + Camre	52	21/31	41/11			0/5/47	17.3%		13.1	
Yang 2022 [60]	TACE + Camre + TKI	31	23/8	27/4	NA	NA	2/18/11	64.5%	8.5	NA	NA
Cai 2022 [61]	TACE + Len + ICIs	41	20/21	37/4	15/26	17/24	NA	56.1%	7.3	16.9	NA
	TACE + Len	40	18/22	33/7	18/22	19/21		32.5%	4.0	12.1	
Teng 2022 [62]	TACE + Len + ICIs	53	35/18	34/19	25/28	42/11	0/23/30	54.9%	8.5	NR	NA
Qu 2022 [55]	TACE + Len + Toripalimab	30	10/20	28/2	27/3	2/28	0/1/29	76.7%	NA	NR	50%
	TACE + Len	21	5/16	21/0	17/4	2/19	0/3/18	47.6%		20.1	19%
Huang 2021 [63]	TACE + Sor + Camre + SBRT	12	4/8	11/1	NA	NA	NA	41.7%	15.7	NR	33.3%

TACE: transarterial chemoembolization, Sor: sorafenib, Len: lenvatinib, Pembro: pembrolizumab, Camre: camrelizumab, TKI: tyrosine kinase inhibitors, SBRT: Stereotactic Body Radiation Therapy, NA: not available, AFP: alpha-fetoprotein, CP: Child Pugh, MVI: macrovascular invasion, EHS: extrahepatic spread, ORR: objective response rate, PFS: progression-free survival, OS: overall survival.

**Table 3:** Ongoing trials evaluating down-staging strategies registered to ClinicalTrial.gov.

Combination Strategies	Trial	Treatment	Phase	Number of pts	Site	Status	Primary Endpoint
*ICI + antiangiogenic (TKI or anti-VEGF)*	NCT04425226 PLENTY202001	Pembrolizumab + Lenvatinib	NA	192	China	Recruiting	RFS
	NCT04443322 Dulect2020-1	Durvalumab + Lenvatinib	NA	20	China	Recruiting	RFS PFS
	NCT05185505	Atezolizumab + Bevacizumab	IV	24	USA	Recruiting	The proportion of Patients Receiving LT Experiencing Acute Rejection
	NCT04035876	Camrelizumab + Apatinib	II	120	China	NA	Objective remission rate, RFS
*ICI doublet*	NCT05027425	Durvalumab + Tremelimumab	II	30	USA	Recruiting	Cellular rejection rates (up to 30 days post-LT)
*TACE +TKI + ICI*	NCT05717738	TACE + TKI + antiPD1	NA	300	China	Recruiting	N. of pts amenable to curative surgical intervention (hepatectomy or LT) or RFA
*TACE + SBRT + ICIs*	NCT03817736	TACE + SBRT + ICIs	II	33	China	Active, not recruiting	N. of pts amenable to curative surgical intervention (hepatectomy or LT)
*TheraSpheres + ICI*	NCT05063565	TheraSphere +/− Durvalumab and Tremelimumab	II	150	USA	Suspended	ORR, DoR
*HAIC + Lenva + antiPD1*	NCT05475613	HAIC + Lenva + antiPD1	II	75	China	Not yet recruiting	2-year RFS rate

TACE: transarterial chemoembolization, HAIC: hepatic arterial infusion chemotherapy, TKI: tyrosine kinase inhibitor, SBRT: Stereotactic Body Radiation Therapy, RFS: relapse-free survival, PFS: progression-free survival, ORR: objective response rate, DoR: duration of response.

## Data Availability

No new data were created or analyzed in this study. Data sharing is not applicable to this article.

## Supplementary Materials

The following supporting information can be downloaded at https://www.mdpi.com/article/10.3390/life13071562/s1, Table S1: Serum half-life of immune-checkpoint inhibitors. References [136,137,138,139,140,141,142,143,144,145] are cited in the supplementary materials.
