# Supplementary material for: Evolving Landscape in Liver Transplantation for Hepatocellular Carcinoma: From Stage Migration to Immunotherapy Revolution
Source: Life (Basel). 2023 Jul 14;13(7):1562. doi: 10.3390/life13071562 (PMC10382048; doi:10.3390/life13071562)
Supplement: Supplementary file 1 [file life-13-01562-s001.zip › life-2425405-supplementary.pdf]

Table S1: Serum half-life of immune-checkpoint inhibitors

ICIs	Mechanism of action	Half-Life	Reference
**Ipilimumab**	Anti CTLA4	14.7 days	[1]
**Tremelimumab**	Anti CTLA4	19.6 days	[2]
**Nivolumab**	Anti PD-1	25 days	[3]
**Pembrolizumab**	Anti PD-1	14-27.3 days	[4]
**Cempiplimab**	Anti PD-1	12 days	[5]
**Toripalimab**	Anti PD-1	14.2 days	[6]
**Sintilimab**	Anti PD-1	35.6 h	[7]
**Avelumab**	Anti PD-L1	3.9-4.1 days	[8]
**Durvalumab**	Anti PD-L1	21 days	[9]
**Atezolizumab**	Anti PD-L1	27 days	[10]

1. Feng, Y.; Masson, E.; Dai, D.; Parker, S.M.; Berman, D.; Roy, A. Model-based clinical pharmacology profiling of ipilimumab in patients with advanced melanoma. Br. J. Clin. Pharmacol. 2014, 78, 106–117.

2. Camacho, L.H.; Antonia, S.; Sosman, J.; Kirkwood, J.M.; Gajewski, T.F.; Redman, B.; Pavlov, D.; Bulanhagui, C.; Bozon, V.A.; Gomez-Navarro, J.; et al. Phase I/II Trial of Tremelimumab in Patients with Metastatic Melanoma. J. Clin. Oncol. 2009, 27,1075–1081.

3. Bajaj, G.; Wang, X.; Agrawal, S.; Gupta, M.; Roy, A.; Feng, Y. Model-Based Population Pharmacokinetic Analysis of Nivolumab in Patients with Solid Tumors. CPT Pharmacomet. Syst. Pharm. 2017, 6, 58–66.

4. Elassaiss-Schaap, J.; Rossenu, S.; Lindauer, A.; Kang, S.P.; de Greef, R.; Sachs, J.R.; de Alwis, D.P. Using Model-Based "Learn and Confirm" to Reveal the Pharmacokinetics-Pharmacodynamics Relationship of Pembrolizumab in the KEYNOTE-001 Trial. CPT Pharmacomet. Syst. Pharm. 2017, 6, 21–28.

5. Papadopoulos, K.P.; Johnson, M.L.; Lockhart, A.C.; Moore, K.; Falchook, G.S.; Formenti, S.C.; Naing, A.; Carvajal, R.D.; Rosen,L.S.; Weiss, G.J.; et al. First-In-Human Study of Cemiplimab Alone or In Combination with Radiotherapy and/or Low-dose Cyclophosphamide in Patients with Advanced Malignancies. Clin. Cancer Res. 2020, 26, 1025–1033.

6. Yang, J.; Dong, L.; Yang, S.; Han, X.; Han, Y.; Jiang, S.; Yao, J.; Zhang, Z.; Zhang, S.; Liu, P.; et al. Safety and clinical efficacy of toripalimab, a PD-1 mAb, in patients with advanced or recurrent malignancies in a phase I study. Eur. J. Cancer 2020, 130, 182–192.

7. Zhang, L.; Mai, W.; Jiang, W.; Geng, Q. Sintilimab: A Promising Anti-Tumor PD-1 Antibody. Front. Oncol. 2020, 10, 594558.

8. Heery, C.R.; O'Sullivan-Coyne, G.; Madan, R.A.; Cordes, L.; Rajan, A.; Rauckhorst, M.; Lamping, E.; Oyelakin, I.; Marté, J.L.; Lepone, L.M.; et al. Avelumab for metastatic or locally advanced previously treated solid tumours (JAVELIN Solid Tumor): A phase 1a, multicohort, dose-escalation trial. Lancet. Oncol. 2017, 18, 587–598.

9. Baverel, P.G.; Dubois, V.F.S.; Jin, C.Y.; Zheng, Y.; Song, X.; Jin, X.; Mukhopadhyay, P.; Gupta, A.; Dennis, P.A.; Ben, Y.; et al. Population Pharmacokinetics of Durvalumab in Cancer Patients and Association with Longitudinal Biomarkers of Disease Status. Clin. Pharmacol. Ther. 2018, 103, 631–642.

10. Stroh, M.; Winter, H.; Marchand, M.; Claret, L.; Eppler, S.; Ruppel, J.; Abidoye, O.; Teng, S.L.; Lin, W.T.; Dayog, S.; et al. Clinical Pharmacokinetics and Pharmacodynamics of Atezolizumab in Metastatic Urothelial Carcinoma. Clin. Pharmacol. Ther. 2017, 102, 305–312.
